# Immediate Skin-to-Skin Contact in Very Preterm Neonates and Early Childhood Neurodevelopment: A Randomized Clinical Trial

**DOI:** 10.1001/jamanetworkopen.2025.5467

**Published:** 2025-04-16

**Authors:** Laila Kristoffersen, Ragnhild Støen, Håkon Bergseng, Silje Tjøm Flottorp, Grete Magerøy, Kristine Hermansen Grunewaldt, Karoline Aker

**Affiliations:** 1 Department of Neonatology, St Olav’s Hospital, Trondheim University Hospital, Trondheim, Norway; 2 Department of Public Health and Nursing, Norwegian University of Science and Technology, Trondheim, Norway; 3 Department of Clinical and Molecular Medicine, Norwegian University of Science and Technology, Trondheim, Norway; 4 Department of Neonatology, Hospital of Southern Norway, Kristiansand, Norway; 5 Department of Neonatology, Drammen Hospital, Drammen, Norway

## Abstract

**Importance:**

Preterm neonates are at risk for neurodevelopmental impairments, and there is a need to identify protective factors that can modify the harmful effects of preterm birth on the immature brain.

**Objective:**

To evaluate whether immediate skin-to-skin contact (SSC) for preterm neonates improves early childhood neurodevelopmental outcomes.

**Design, Setting, and Participants:**

This open-label randomized clinical trial was conducted in 3 Norwegian neonatal units between February 2014 and October 2020. Participants were preterm neonates born at 28 weeks 0 days’ to 31 weeks 6 days’ gestation with birth weight greater than 1000 g and no major congenital malformations or need for intubation or oxygen supplementation of more than 40%. Intention-to-treat analysis was conducted from July 2023 to July 2024.

**Intervention:**

Neonates were randomized 1:1 to immediate SSC between mother and neonate in the delivery room for 2 hours or to standard care with direct transport to the neonatal unit in an incubator.

**Main Outcomes and Measures:**

The primary outcome was cognitive development at 2 to 3 years of age, measured by the Bayley Scales of Infant and Toddler Development, Third Edition (BSID-III). Secondary outcomes were language and motor neurodevelopment measured by the BSID-III, parental questionnaires at 3 and 12 months and 2 to 3 years, and breastfeeding practices up to 12 months.

**Results:**

Of 108 included neonates (68 [63%] male; mean [SD] gestational age, 30 weeks 3 days [1 week 1 day]), 51 received SSC and 57 received standard care. Eighty-six (80%) had follow-up at 2 to 3 years, and 81 (75%) completed the BSID-III and were analyzed for the primary outcome. The mean difference in BSID-III cognitive composite scores was 0.21 (95% CI, −5.26 to 5.68; *P* = .94). There was no difference between the groups in the proportion at risk of developmental delay at 2 to 3 years: 21 of 41 (51%) and 22 of 45 (49%) in the SSC and standard care groups, respectively (odds ratio, 1.10 [95% CI, 0.47-2.56]; *P* = .83). More neonates in the SSC group were breastfed at hospital discharge (42 of 50 [84%] vs 36 of 54 [67%]; *P* = .04).

**Conclusions and Relevance:**

In this randomized clinical trial, 2 hours of mother-neonate SSC in the delivery room did not enhance neurodevelopmental outcomes at 2 to 3 years of age. However, the SSC group demonstrated improved breastfeeding practices up to 12 months compared with standard care, suggesting that the feasible and low-cost SSC intervention should be encouraged in clinical practice.

**Trial Registration:**

ClinicalTrials.gov Identifier: NCT02024854

## Key Points

**Question:**

Does skin-to-skin contact (SSC) between very preterm neonates and their mothers immediately after delivery affect neurodevelopmental outcomes in early childhood?

**Findings:**

In this randomized clinical trial of 108 neonates born at 28 weeks 0 days’ to 31 weeks 6 days’ gestation and randomized to immediate SSC or standard care in an incubator, no difference in neurodevelopmental outcomes at age 2 to 3 years was found.

**Meaning:**

These findings suggest that immediate SSC after delivery for very preterm neonates does not affect neurodevelopment at age 2 to 3 years.

## Teaser

This randomized clinical trial evaluates whether direct skin-to-skin contact between very preterm neonates and their mothers immediately after delivery vs standard care in an incubator improves neurodevelopmental outcomes at 2 to 3 years of age.

## Introduction

Long-term health issues are still a major problem for many individuals born preterm.^1,2^ Compared with full-term neonates, very preterm neonates (28-31 weeks’ gestation) are at increased risk of long-term impairments such as cerebral palsy; motor, cognitive, and language delay; behavioral problems; psychiatric disorders; and visual and hearing impairments.^1,3,4,5,6^ The mean IQ score in very preterm infants is approximately 1 SD lower than in infants born full-term.^4^ Numerous early intervention programs have been developed to compensate for the negative consequences of preterm birth.^7,8^ One intervention is kangaroo mother care (KMC),^9^ in which skin-to-skin contact (SSC) is the primary component.^10^ SSC between a caretaker and a preterm newborn in the neonatal intensive care unit (NICU) is associated with reduced neonatal morbidities and parental stress and with improved parental mental health.^11,12,13^

The first hours after birth are considered an early sensitive period that is important for mother-neonate interaction and secure attachment.^14,15,16^ This has led to an increased focus on a nonseparation approach and immediate SSC following delivery between preterm neonates and their mothers or caregivers. The World Health Organization recently amended its recommendations for the care of preterm infants to advise immediate SSC “as soon as possible after birth.”^10^ This recommendation is based on a randomized clinical trial (RCT) including more than 3000 low-birth-weight infants in low-resource settings and reporting reduced mortality in infants receiving SSC from birth.^17^ Studies from low- and high-resource settings have shown improved thermoregulation and cardiorespiratory stability in preterm neonates receiving immediate SSC.^18,19,20,21,22,23^ Studies have also reported less maternal depression; reduced feelings of fear, guilt, and frustration; and a higher breastfeeding rate with immediate SSC between mother and newborn.^24,25,26^ The nonseparation approach and immediate SSC following delivery is a paradigm shift in neonatal medicine. Yet, few hospitals facilitate immediate SSC, and no studies to our knowledge have reported an association between delivery room SSC and early childhood outcomes.

We conducted an RCT comparing 2 hours of delivery room SSC between very preterm neonates and their mothers vs standard care. The primary aim of this study was to investigate whether immediate SSC improves cognitive outcomes at 2 to 3 years.^27^ Safety and feasibility have been reported elsewhere.^19^

## Methods

### Study Design and Setting

This was a prospective, multicenter, open-label RCT comparing 2 hours of early SSC between mothers and very preterm neonates with standard care in an incubator (NCT02024854). The original trial protocol is provided in Supplement 1. The study was initiated at St Olav’s Hospital, Trondheim University Hospital (Trondheim, Norway) in February 2014. In January 2017, 2 additional Norwegian hospitals (Drammen Hospital, Drammen, and Hospital of Southern Norway, Kristiansand) started recruiting participants. The Regional Committee for Medical and Health Research Ethics in central Norway approved the trial. Written informed consent was obtained from participating mothers and their partners. This study followed the Consolidated Standards of Reporting Trials (CONSORT) reporting guideline for RCTs.

### Participants

Between February 2014 and October 2020, a dedicated neonatal nurse or physician approached and informed pregnant patients admitted due to the risk of preterm birth between pregnancy weeks 28 and 31. Neonates were included if born at a gestational age of 28 weeks 0 days to 31 weeks 6 days and with a birth weight of at least 1000 g. Exclusion criteria were the need for more than 40% oxygen to maintain oxygen saturation of greater than 90% at 20 minutes of life, the need for intubation and mechanical ventilation in the delivery room, major congenital malformations, and maternal general anesthesia.

### Randomization and Intervention

Eligible pregnant patients and their partners received information about the study and completed written informed consent before the delivery. Following delivery and cord clamping, baseline assessment and screening were done by the neonatal delivery room team. Included neonates were randomized in a 1:1 ratio to 2 hours of mother-neonate SSC in the delivery room or standard care with transfer to the NICU in an incubator. After the attending physician decided on the neonate’s eligibility, randomization was done by the neonatal nurse in charge using sealed envelopes stratified by center and gestational age (28 weeks 0 days to 29 weeks 6 days or 30 weeks 0 days to 31 weeks 6 days) with variable block sizes. Thereafter, the physician or the nurse allocated the neonate to the designated intervention arm. During SSC, the neonate was placed in an upright position between the mother’s breasts, with arms and legs flexed. The head was slightly elevated to 1 side to secure airways. Blinding was not possible due to the nature of the intervention. A recent publication from the same cohort provides a more detailed description of the study participants and the intervention.^19^

### Breastfeeding and Growth

Data on the amount of breastfeeding at NICU discharge were extracted from the neonate’s medical record. Information about breastfeeding and breast milk up to 1 year of age was obtained from a parental questionnaire comprising 8 selected questions from a questionnaire by the Norwegian Directorate of Health.^28^ Growth was calculated by PediTools Growth Parameters based on the 2013 Fenton growth charts. Small for gestational age is defined as birth weight below the 10th percentile and large for gestational age as birth weight above the 90th percentile.^29,30^ Data were collected prospectively.

### Neurodevelopmental Follow-Up

After discharge, infants were followed up at 3 months, 12 months, and 2 to 3 years of corrected age (the age calculated from the due date at ultrasonography). When reporting age, we refer to the corrected age unless otherwise specified. An overview of the outcome assessments is presented in the eFigure in Supplement 2. At 10 to 16 weeks of age, infants were videorecorded for 3 to 5 minutes for the General Movement Assessment (GMA), and motor development was evaluated with the Motor Optimality Score–Revised (MOS-R).^31^ The videorecordings were performed and classified according to the Prechtl method.^32^ The MOS-R and the presence or absence of fidgety movements are used to identify infants at risk of adverse developmental outcomes.^32^ Certified GMA observers were blinded to the intervention. The MOS-R score ranges from 5 to 28, where a score of 25 to 28 is considered optimal; 20 to 24 indicates the child’s motor repertoire is mildly reduced; 9 to 19, moderately reduced; and 5 to 8, severely reduced.^31^

Two parent-completed questionnaires, the Ages & Stages Questionnaire (ASQ) and the ASQ–Social-Emotional (ASQ-SE), were completed at the following time points: 3 months (only ASQ-SE), 12 months, and 2 to 3 years of age. The ASQ is a questionnaire evaluating the child’s development in 5 domains: communication, problem solving, fine motor, gross motor, and personal-social.^33^ A Norwegian version of this screening tool has been validated as an appropriate assessment for evaluating children’s development.^34^ The scores are summed to give scores for each subscale and a total sum score. In this study, risk of developmental delay was defined as a score below the cutoff in the Norwegian ASQ manual.^35^ There are different age-dependent cutoff scores for all domains. The child’s socioemotional and behavioral difficulties and competencies were assessed using the ASQ-SE^36^ The domains assessed are self-regulation, compliance, communication, adaptive functioning, autonomy, affect, and interaction with people. Scores are summed to give a total sum score. This screening instrument is valid in identifying social and emotional difficulties in children.^37^ Risk of developmental delay was defined as a score above the age-dependent cutoff score in the Norwegian ASQ-SE manual.^38^

At 12 months and 2 to 3 years of age, neurodevelopmental outcomes were assessed with the Bayley Scales of Infant and Toddler Development, Third Edition (BSID-III).^39^ The BSID-III comprises 3 developmental domains: cognitive, language, and motor. Each domain has a normed score (composite score) with a mean score of 100 and an SD of 15. Higher scores indicate development consistent with normative data.^39^ We defined risk of developmental delay as a composite score below 90 in any domain. This is the clinical cutoff used in the Swedish neonatal guidelines based on assessments of healthy controls born early-, full-, or late-term in the Extremely Preterm Infants in Sweden Study (EXPRESS).^40,41^ Two licensed practitioners blinded to the intervention conducted the assessments. After 2 years of enrollment, the 12-month BSID-III assessment was replaced with the ASQ and ASQ-SE. This was due to limited staff resources to conduct the testing and to reduce the burden of physical follow-up assessments on the families. The last follow-up was planned at 2 years of age, but due to the COVID-19 pandemic, some participants had their follow-up postponed up to age 3 years.

### Outcomes

The primary outcome of this study was BSID-III cognitive composite score at 2 to 3 years. BSID-III language and motor composite scores were secondary outcomes. Other secondary outcomes were ASQ-SE and GMA at 3 months, ASQ-SE and ASQ or BSID-III at 12 months, ASQ and ASQ-SE at 2 to 3 years, and breastfeeding practices up to 12 months.

### Statistical Analysis

The sample size was calculated to detect a mean (SD) difference of 7.5 (15.0) in BSID-III cognitive composite scores at 2 years (α = .05; β = 0.08).^27^ Allowing for 6% loss to follow-up, the total sample size needed was 136 neonates. Based on the number of very preterm births at the 3 collaborating hospitals, the sample size was expected to be achieved by the end of 2018. After 6.5 years, inclusion was stopped after enrollment of 108 neonates on October 1, 2020.

Analysis was by intention to treat. Demographic factors and clinical characteristics were summarized with counts (percentages) for categorical variables and means (SDs) or medians (IQRs) for continuous variables. Differences between the SSC and standard care groups for continuous variables were analyzed using 2-sample *t* tests or Mann-Whitney *U* tests, as appropriate. Categorical variables were analyzed by the Pearson χ^2^ test, Fisher exact test, or linear-by-linear associations. Two-sided *P* < .05 was considered statistically significant. The primary outcome was presented as an unadjusted mean difference with a comparison between groups using the 2-sample *t* test. The adjusted mean difference was calculated using linear regression adjusting for the neonate’s sex and maternal educational level of a bachelor’s degree or higher. We summarized children at risk of developmental delay in any of the assessments (ASQ, ASQ-SE, and BSID-III) at 2 to 3 years, with a comparison between the groups using Pearson χ^2^ test or Fisher exact test. Results are reported as unadjusted odds ratios (ORs). Adjustments for child’s sex and maternal educational level did not affect the results and are not reported. The statistical analysis was conducted from July 2023 to July 2024 using SPSS, versions 28 and 29 (IBM Corp).

## Results

### Study Participants

A total of 108 neonates (mean [SD] gestational age, 30 weeks 3 days [1 week 1 day]; 40 females [37%] and 68 males [63%]) of 101 mothers were included in the study and randomly assigned to either immediate SSC (n = 51) or standard care (n = 57). Twenty-two neonates (20%) were lost to follow-up; hence, 86 (80%) had any or some follow-up at 2 to 3 years of age (BSID-III, ASQ, ASQ-SE, or any 2 or 3 of these assessments) (Figure and eFigure in Supplement 2). Eighty-six children (80%) met for BSID-III assessment at 2 to 3 years, but assessments for 5 of those children (6%) could not be evaluated due to no BSID-III or the child’s inability to test. Therefore, 81 children (75%) were analyzed for the primary outcome (39 [76%] in the SSC group and 42 [74%] in the standard care group). Maternal and neonatal characteristics at birth are summarized according to the intervention group in Table 1 for all participants and in eTable 1 in Supplement 2 for participants with the primary outcome.

**Figure.**
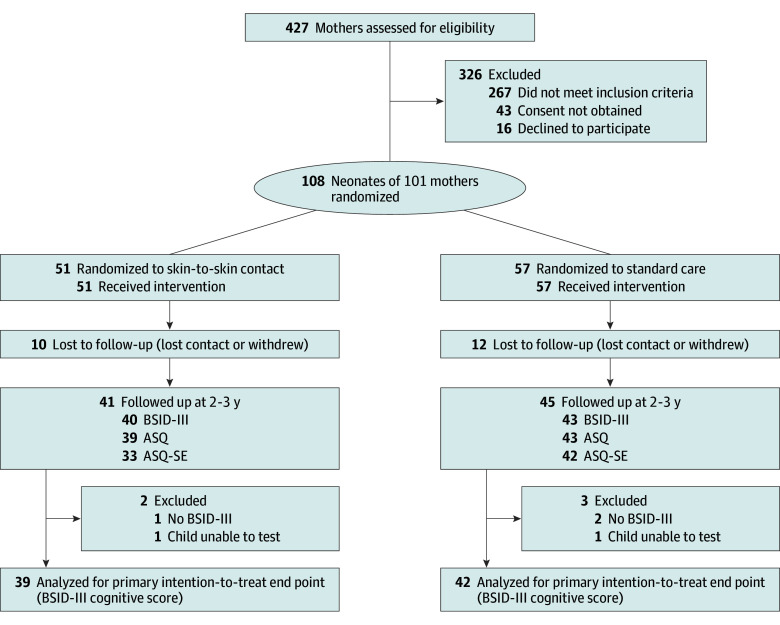
Study Flow Diagram ASQ indicates Ages & Stages Questionnaire; ASQ-SE, Ages & Stages Questionnaire–Social-Emotional; BSID-III, Bayley Scales of Infant and Toddler Development, Third Edition.

**Table 1.** Maternal and Neonatal Characteristics According to Intervention Group

Characteristic	Participants			
	Skin-to-skin contact (n = 51)		Standard care (n = 57)	
	Total No.	No. (%)^a^	Total No.	No. (%)^a^
**Maternal**				
Age at delivery, mean (SD), y	45	32.2 (5.0)	52	30.7 (5.9)
University education^b^	45	32 (71)	48	27 (56)
Cesarean delivery	47	27 (57)	54	31 (57)
Multiple birth	47	4 (9)	54	3 (6)
**Neonatal**				
Gestational age, mean (SD), wk	51	30.3 (1.1)	57	30.3 (1.2)
Birth weight, mean (SD), g	51	1436 (266)	57	1438 (257)
Sex				
Female	51	18 (35)	57	22 (39)
Male	51	33 (65)	57	35 (61)
Growth^c^				
Small for gestational age	51	1 (2)	57	1 (2)
Large for gestational age	51	2 (4)	57	3 (5)

^a^ Data are presented as number (percentage) of participants unless otherwise indicated.

^b^ Completed a bachelor’s degree or higher.

^c^ Calculated by PediTools growth parameters based on the 2013 Fenton growth charts. Small for gestational age is defined as birth weight below the 10th percentile and large for gestational age as birth weight above the 90th percentile.^29,30^

### Primary Outcome

The median age at BSID-III was 24 months (IQR, 24-25 months) in the SSC group and 25 months (IQR, 24-26 months) in the standard care group. There was no significant difference between groups in the primary outcome, BSID-III cognitive composite score (mean difference, 0.21; 95% CI, −5.26 to 5.68; *P* =  .94) (Table 2).

**Table 2.** BSID-III, ASQ, and ASQ-SE Scores at 2 to 3 Years of Corrected Age

Measure	Skin-to-skin contact		Standard care		MD (95% CI)	*P* value	AMD (95% CI)^a^	*P* value
	Participants, No.	Score	Participants, No.	Score				
**Primary outcome**								
BSID-III composite— cognitive, mean (SD)	39	99.6 (11.1)	42	99.4 (13.4)	0.21 (−5.26 to 5.68)	.94	0.31 (−5.13 to 5.76)	.91
**Secondary outcomes**								
BSID-III composite, mean (SD)								
Language	39	97.7 (14.4)	42	95.4 (12.8)	2.33 (−3.67 to 8.34)	.44	−1.44 (−7.42 to 4.54)	.63
Motor	39	95.7 (9.5)	42	97.4 (14.9)	−1.71 (−7.30 to 3.87)	.54	2.87 (−2.66 to 8.39)	.30
ASQ, median (IQR)^b^^,^^c^								
Gross motor	39	60 (50-60)	43	55 (45-60)	NA	.08^d^	NA	NA
Fine motor	39	53 (50-60)	43	55 (50-60)	NA	.50^d^	NA	NA
Problem-solving	39	50 (40-55)	43	50 (40-55)	NA	.83^d^	NA	NA
Communication	39	55 (45-60)	43	50 (45-60)	NA	.30^d^	NA	NA
Personal-social	39	50 (40-55)	43	50 (40-55)	NA	.48^d^	NA	NA
ASQ-SE, median (IQR)^b^^,^^e^	33	20 (13-40)	42	23 (10-38)	NA	.98^d^	NA	NA

Abbreviations: AMD, adjusted mean difference; ASQ, Ages & Stages Questionnaire; ASQ-SE, Ages & Stages Questionnaire–Social-Emotional; BSID-III, Bayley Scales of Infant and Toddler Development, Third Edition; MD, mean difference; NA, not applicable.

^a^ Adjusted for sex and maternal university education (completion of a bachelor’s degree or higher).

^b^ ASQ and ASQ-SE scores are not normally distributed; thus, data were analyzed using the Mann-Whitney *U* test, and MDs and AMDs are not reported.

^c^ Higher ASQ scores indicate better development.

^d^ *P* value from Mann-Whitney *U* test.

^e^ Lower ASQ-SE scores indicate better development.

### Secondary Outcomes

At 2 to 3 years, 21 of 41 children (51%) in the SSC group and 22 of 45 (49%) in the standard care group were classified as at risk of developmental delay on either the BSID-III, ASQ, or ASQ-SE, with no difference between the groups in the proportion of infants at risk of developmental delay (OR, 1.10; 95% CI, 0.47-2.56; *P* = .83) (Table 3). Ninety-two infants (85%) returned for follow-up at 3 months and 88 (81%) at 12 months (eFigure and eTable 2 in Supplement 2). In the standard care group, 1 infant (2%) with absent fidgety movements was lost to follow-up after 3 months. No infants had severely reduced MOS-R scores, and 3 infants in each group had moderately reduced MOS-R scores. Infants in the SSC group had significantly lower scores in the personal-social domain of the ASQ compared with infants in the standard care group at 12 months (median, 35 [IQR, 30-45] vs 45 [IQR, 40-55]; *P* = .02) (eTable 1 in Supplement 2), but this difference was not present at 2 to 3 years (Table 2).

**Table 3.** ORs for Surpassing the Clinical Cutoff on BSID-III, ASQ, and/or ASQ-SE at 2 to 3 Years of Corrected Age

Measure	Participants, No./total No. (%)		OR (95% CI)	*P* value
	Skin-to-skin contact	Standard care		
BSID-III below clinical cutoff (<90)				
Cognitive composite score	4/39 (10)	5/42 (12)	0.85 (0.21-3.41)	>.99
Language composite score	10/39 (26)	11/42 (26)	0.97 (0.36-2.63)	.96
Motor composite score	11/39 (28)	11/42 (26)	1.11 (0.42-2.95)	.84
Any composite score	15/39 (38)	15/42 (36)	1.12 (0.46-2.77)	.80
ASQ score below clinical cutoff^a^				
Gross motor	3/39 (8)	4/43 (9)	0.81 (0.17-3.88)	>.99
Fine motor	2/39 (5)	3/43 (7)	0.72 (0.11-4.56)	>.99
Problem-solving	4/39 (10)	1/43 (2)	4.80 (0.51-44.94)	.19
Communication	2/39 (5)	5/43 (12)	0.41 (0.07-2.25)	.44
Personal-social	1/39 (3)	5/43 (12)	0.20 (0.02-1.79)	.21
Any	9/39 (23)	11/43 (26)	0.87 (0.32-2.40)	.79
ASQ-SE score below clinical cutoff^b^	3/33 (9)	8/42 (19)	0.42 (0.10-1.75)	.33
BSID-III, ASQ, and/or ASQ-SE score below clinical cutoff	21/41 (51)	22/45 (49)	1.10 (0.47-2.56)	.83

Abbreviations: ASQ, Ages & Stages Questionnaire; ASQ-SE, Ages & Stages Questionnaire–Social-Emotional; BSID-III, Bayley Scales of Infant and Toddler Development, Third Edition; OR, odds ratio.

^a^ Clinical cutoff was according to the Norwegian ASQ manual.^35^

^b^ Clinical cutoff was according to the Norwegian ASQ-SE manual.^38^

More neonates in the SSC group were breastfed at discharge (42 of 50 [84%], vs 36 of 54 [67%] in standard care; *P* = .04) (Table 4). The duration of exclusive breastfeeding was longer in the SSC group, and at 12 months, 18 of 41 (44%) in the SSC group and 11 of 42 (26%) in the standard care group were breastfed (*P* = .07).

**Table 4.** Breastfeeding Outcomes From Discharge to 12 Months of Corrected Age

Outcome	Participants, No./total No. (%)		*P* value
	Skin-to-skin contact	Standard care	
**At discharge**			
Breastfeeding			
Any	42/50 (84)	36/54 (67)	.04
Exclusive	32/50 (64)	26/54 (48)	.10
Breast milk proportion per daily feed, %			
>75	36/41 (88)	31/44 (70)	.01
25-75	4/41 (10)	2/44 (5)	
<25	1/41 (2)	11/44 (25)	
**At 12 mo**			
Duration of any breastfeeding, mo			
0 to <3	3/41 (7)	7/42 (17)	.03
3 to <6	7/41 (17)	10/42 (24)	
6 to <9	6/41 (15)	10/42 (24)	
9 to <12	7/41 (17)	4/42 (10)	
≥12	18/41 (44)	11/42 (26)	
Duration of exclusive breastfeeding, mo			
0 to <3	7/38 (18)	11/38 (29)	.03
3 to <6	12/38 (32)	19/38 (50)	
≥6	19/38 (50)	8/38 (21)	

## Discussion

In this RCT comparing delivery room SSC with standard care in an incubator for very preterm neonates, we found no significant or clinically relevant differences between groups in neurodevelopmental outcomes at 2 to 3 years of age. There were also no differences in neurodevelopmental outcomes between groups at 3 and 12 months. However, significantly more neonates in the SSC group were breastfed at discharge compared with those in the standard care group. This difference persisted up to 1 year of age.

To our knowledge, this is the first RCT reporting early childhood outcomes after delivery room SSC for very preterm neonates. An RCT in Columbia reported long-term outcomes of continuous KMC initiated after a median of 4 days after birth vs traditional care.^42,43^ It found no difference between groups at 6 and 12 months, but in a subset of the study population assessed at 20 years of age, there was a dose-dependent positive association between the duration of KMC and the volume of certain brain structures measured with magnetic resonance imaging. The clinical significance of such a finding is not clear.

Improved bonding and maternal-infant relationship have been demonstrated at 4 and 6 months of age, respectively, in preterm neonates exposed to immediate SSC after delivery.^25,44^ Studies also report that a longer duration of KMC in the NICU improves infant growth, breastfeeding, and neurobehavioral performance.^45,46^ No early childhood or long-term follow-up is available from these studies. However, results are difficult to compare for all outcomes, as there are various definitions of early or immediate KMC and SSC (from immediately after delivery to several days after birth) as well as differences in gestational age groups included and how data on duration of KMC and SSC are retrieved.^17,18,19,20,23,25^

Our results are similar to those of a recent RCT on music therapy intervention in the NICU for preterm neonates (the LongSTEP trial), reporting no difference between groups in BSID-III composite score at 2 years.^47^ It has been difficult to demonstrate improved long-term benefits after both music therapy and SSC despite short-term improvements in physiological stability and reduced maternal stress and anxiety shown in smaller studies.^19,20,23,25,47^ Very preterm infants in high-income settings have high survival rates, and almost 80% do not have motor or cognitive delays.^48^ In our study, the mean BSID-III composite scores at 2 to 3 years were within what is considered the normal range. However, both groups had a lower mean composite score on all BSID-III domains compared with 633 healthy full-term infants who served as a control group in the Swedish EXPRESS.^40^ Few infants included in our study had severe complications of prematurity, and there were no differences in complication rates between the groups.^19^ Our results suggest that in this high-resource context where most infants experience normal neurodevelopment, single interventions like SSC may not influence hard end points like survival and BSID-III scores at 2 to 3 years.

The present study showed that more neonates in the SSC group were breastfed at discharge, and the duration of exclusive breastfeeding was longer than among neonates receiving standard care. These findings are in accordance with another study on immediate SSC for preterm neonates reporting a higher, although nonsignificant, incidence of exclusive breastfeeding at discharge in the SSC group.^25^ A recent study reported that close contact between the mother and neonate within 30 minutes after birth and regular SSC between mother and child during the NICU stay were associated with successful breastfeeding in moderate- to late-preterm infants.^49^ This finding was supported by a meta-analysis including 1900 preterm and low-birth-weight infants that demonstrated that KMC was associated with reduced breastfeeding initiation time compared with conventional care.^50^ Although we did not find an association of immediate SSC with neurodevelopment, a recent Swedish study reported that not receiving breast milk at discharge from the NICU was 1 of the 5 most important features of cognitive delay in very preterm infants.^51^ The associations between SSC, breastfeeding, nutritional status, socioeconomic and societal conditions, and cognitive outcomes in preterm infants are impossible to explore in small studies like ours and may be difficult to assess even in larger studies. We do not believe that the lack of association between immediate SSC and neurodevelopmental outcomes should be used as an argument against the positive changes that have occurred in neonatal care over the past decades. On the contrary, focus on family-integrated care, closeness between parents and infant, and empowering of parents in their caretaking role must be considered essential to optimize and promote a nurturing environment for preterm infants and their parents.

### Limitations

Our study has important limitations. By protocol, the sickest neonates were excluded since this was our first attempt to provide SSC in the delivery room for very preterm neonates, and safety concerns were important to address. We also did not collect data on the total duration of SSC during the hospital stay. After 6 years of recruitment, the estimated sample size of 136 was not reached, and we found it unethical to continue randomization and consequently hinder immediate mother-neonate SSC. However, with almost 80% of the planned sample size included, there were no trends in any direction, and we find it unlikely that a slightly larger sample size would have changed our conclusions. Another limitation was the follow-up process. We underestimated the challenge of repeated follow-up appointments. The COVID-19 pandemic and the inclusion of more study sites also added to the complexity of follow-up. The lack of a healthy, full-term–born control group was also a limitation, as we do not have Norwegian norms for the BSID-III. However, the control group of 366 healthy full-term infants in EXPRESS was considered suitable.^40^ In this study, socioeconomic status was assessed solely based on maternal educational level, as these were the only data available. Additional information on socioeconomic status, educational level, and the home environment would have been beneficial. Additionally, we had limited data on markers of neonatal illness.^19^ Since the available data showed no difference between groups, we did not include this in the adjusted outcome analyses.

## Conclusions

In this RCT, 2 hours of mother-neonate SSC in the delivery room did not improve neurodevelopmental outcomes at 2 to 3 years of age. Significantly more neonates in the SSC group were breastfed at discharge and up to 1 year of age compared with those in the standard care group. Although immediate SSC did not translate into improved neurodevelopment, a nonseparation approach might still have an impact on breastfeeding outcomes. Instead of doing more studies on immediate SSC, resources should be focused on implementing this important, feasible, and low-cost intervention.

## Notes

Supplement 1.
Trial Protocol

Supplement 2. **eFigure.** Overview of Outcome Assessments at 3 and 12 Months and 2 to 3 Years of Corrected Age **eTable 1.** Maternal and Neonatal Characteristics Among Infants With the Primary Outcome, According to Intervention Group **eTable 2.** Neurodevelopmental Outcomes at 3 and 12 Months of Corrected Age
eReferences.

Supplement 3.
Data Sharing Statement
