# Supplementary material for: Immediate Skin-to-Skin Contact in Very Preterm Neonates and Early Childhood Neurodevelopment: A Randomized Clinical Trial
Source: JAMA Netw Open. 2025 Apr 16;8(4):e255467. doi: 10.1001/jamanetworkopen.2025.5467 (PMC12004208; doi:10.1001/jamanetworkopen.2025.5467)
Supplement: Supplement 1. — Trial Protocol [file jamanetwopen-e255467-s001.pdf]

This supplement contains the following:

1. Original protocol.
2. Summary of amendments to the original protocol.
3. Summary of amendments to the published protocol.
4. Ethical approval document after amendments.

1. Original protocol

Skin-to-skin After Delivery in Preterm Infants Born at 28-32 Weeks of Gestation. A Randomized Controlled Trial

Background

Worldwide, 15 million infants are born preterm annually (1), and preterm birth is one of the largest direct causes of neonatal mortality and morbidity (2). Compared to full term infants, preterm infants are at increased risk of neurodevelopmental impairments (3-5). This also includes lower self-esteem, social relations and quality of life in adulthood (6). There is growing evidence that mental and behavioral problems in children born preterm last into adulthood (7-13). These societal and medical consequences of preterm births have led to a growing interest in developmental care models to optimize neurodevelopmental outcomes. Different approaches aiming to support infants and parents have been established in neonatal intensive care units (NICUs) in recent years, and some of these early intervention programs seem to have a positive effect on long-term function up to pre-school age (5, 14, 15). The first hours after birth represent a sensitive period for the very low birthweight infant (VLBW), and mothers who see their infant within three hours after birth are likely to establish a more secure attachment to the infant compared to those who do not see their infant within three hours (16). Parents are increasingly acknowledged as primary caregivers for their preterm born infant; despite the need for intensive care, most NICUs try to facilitate early parent-infant bonding. Education of parents to understand subtle signs and signals from their tiny, preterm-born infants is considered an investment in an optimal home environment for the child. From being forced to separate from their newborn infant due to restrictions of visiting hours in NICUs, parents are now generally encouraged to stay with their infant as much as possible (17, 18).

Kangaroo Care (KC) (19), Family-Centered Care (FCC) (20), Newborn Individualized Development Care and Assessment Program (NIDCAP) (21, 22) and a variety of early intervention programs emphasize the importance of establishing early parent-infant interaction to support an optimal development (23-25). Facilitating early skin-to-skin care

(SSC) is one way of supporting early parent-infant bonding and is also associated with improved physiological stability (26, 27) and decreased cortisol reactivity (28). In addition, a recent WHO guideline on interventions to improve outcomes for preterm infants, strongly recommends early SSC as thermal care for preterm infants weighing <2000 grams (29).
Skin-to-skin care in the delivery room (DR) has been studied for preterm infants from 32 weeks of gestation (30). However, to the best of our knowledge, SSC in the DR and the operating room (OR) has not been systematically investigated for infants born at gestational age (GA) <32 weeks. Obvious obstacles to do so would be the need for medical equipment and competent personnel, usually available only in the NICU. Breathing support, need of surfactant, intravenous access and monitoring equipment are needed in a large proportion of infants below 32 weeks. To combine medical interventions with early SSC, a trained team and formalized guidelines are required to ensure the medical safety of the infant. Specific challenges arise with early SSC in the OR after cesarean section (C-section).
In the present study, we aim to investigate whether SSC following delivery is safe, and how it affects early and late outcomes compared to standard care for preterm infants born at GA 28^0 – 31^6 weeks.

Aims

Primary aim

To study the effect of early SSC versus standard care (SC) for very preterm infants (28^0 – 31^6 weeks) on cognitive scores at two years corrected age, measured with Bayley Scales of Infant Development, Third Edition (Bayley III).

Secondary aims

- To assess safety of the intervention by measuring incidence of hypothermia (temperature <36°C) or respiratory failure during the first 2 hours of life.
- To estimate the effect of the intervention on complications to prematurity (intraventricular hemorrhage (IVH) or periventricular leukomalacia (PVL), seizures, necrotizing enterocolitis (NEC), treatment for persistent ductus arteriosus (PDA) and bronchopulmonary dysplasia (BPD)).
- To estimate the effect of the intervention on physiological stability during the first 24 hours.

- To investigate if the intervention affects the general movements and detailed aspects of the early motor repertoire within 24 hours after birth and at corrected age three months, using the General Movement Assessment (GMA) and the Assessment of Motor Repertoire.
- To investigate the effect of the intervention on social and emotional competence at corrected age three months with The Ages and Stages Questionnaire- Social Emotional.
- To estimate the effect of the intervention on neurodevelopmental outcomes including language and motor function at corrected age two years assessed with Bayley III.

Methods/Design

Project context

The study will be carried out at St. Olavs University Hospital in Trondheim. Approximately 50 infants with GA <32 weeks are born every year at St. Olavs Hospital.

Study design

The study is designed as an RCT with randomization to skin-to-skin care (SSC) or standard care (SC) in an incubator after delivery.

Study population

Singleton and twin preterm infants with GA 28^0-31^6 and a BW>1000 grams in a stable medical condition delivered either vaginally or by C-section are candidates for inclusion. Twins are randomized to the same intervention.
Infants who need intubation and mechanical ventilation, or CPAP with more than 40% oxygen to maintain a saturation above 90% at 20 minutes of age, are excluded. Mothers have to be awake (not under general anesthesia) during C-section.

Intervention

For all infants of eligible women, cord milking is advised immediately following delivery and before the infant is placed on the resuscitation unit. Predefined blood samples are taken from the umbilical cord. Blood gas is taken from the umbilical artery before cord clamping, while blood for hemoglobin, leukocytes, thrombocytes, CRP and blood typing is sampled after the cord clamping. After the initial stabilization, eligibility is decided by the consultant. Infants randomized to standard care are transferred to the NICU in an incubator after stabilization.

The father will usually follow the newborn to the NICU, while the mother will come as soon as possible after delivery. All infants with a BW <1500g, irrespective of randomization, are given parenteral nutrition (glucose and amino acids) after the initial stabilization. Infants with a GA<30 weeks receive CPAP, infants ≥30 weeks get CPAP if clinically indicated. If necessary, surfactant can be administered via a thin catheter in the trachea during spontaneous breathing on CPAP ("surfactant without intubation" (SWI) if oxygen requirement exceeds 35% on CPAP and/or the infant has signs of moderate to severe respiratory distress. After the infant is stabilized in skin-to-skin position on the mother's chest (SSC) or in a closed incubator and transferred to the NICU (SC), the infant is evaluated according to a checklist. Infants in the intervention group are offered SSC up to two hours after delivery – in the delivery room after vaginal delivery, or in the recovery room after C-section. A consultant and a neonatal nurse are responsible for the infant, while a midwife takes care of the mother. After C-section, the same personnel from the NICU and from the maternity ward are present in addition to specialist nurses from the postoperative ward. When the infant is stabilized on the mother's chest, the consultant can leave the DR or postoperative ward in agreement with the neonatal nurse in charge. The consultant is nearby and easily reachable by telephone.

Further treatment during the hospitalization does not differ between groups. According to the unit's guidelines, both mothers and fathers have unrestricted access to the NICU and are encouraged to have as much skin-to-skin contact with their preterm infant as possible.

Outcomes measures

Primary outcome

Bayley Scales of Infant and Toddler Development, Third Edition (Bayley III)

The Bayley-III is a screening test that includes assessment of cognition, language (receptive and expressive), and motor function (gross and fine) in infants and young children from 0 to 42 months and provides a measurable and validated cognitive quotient (31).

Secondary outcomes

Hypothermia (<36°C), respiratory failure requiring acute intubation and/or Cardiopulmonary Resuscitation (CPR). Physiological stability. Blood glucose. Physiological variables are recorded during the first 24 hours.

General Movement Assessment (GMA)

All infants are videotaped within 24 hours after birth (preterm movements period) and at 10-15 weeks' post term age (Fidgety Movements' period) for the GMA and the AMR. The video recordings are performed and classified according to the Prechtl method (32, 33) by a certified GMA observer unaware of the intervention group.

Ages & Stages Questionnaire-Social-Emotional (ASQ-SE)

The child's social and emotional competence is evaluated using the ASQ-SE at corrected age three months and two years (34). The ASQ-SE comprises 22 questions which address seven behavioral areas: self-regulation, compliance, communication, adaptive functioning, autonomy, affect, and interaction with people. The ASQ-SE is a screening instrument developed for children aged three months to five years.

Clinical registrations

A Case Report Form (CRF) has been prepared in cooperation with the Unit for Applied Clinical Research at the Norwegian University of Science and Technology (NTNU). The CRF is divided into three parts.

On admission and the first 24 hours:

Background data which are recorded are: mode of delivery, GA, sex, Apgar scores at one, five and ten minutes, birthweight, length, head circumference, maternal cause of preterm delivery (preeclampsia, breech position, rupture of membranes, premature contractions or infection), fetal cause of preterm delivery (growth retardation, non-reassuring CTG registration) and antenatal steroids (full or incomplete course).

Observations and interventions which are recorded during the first 24 hours are: oxygen saturation and requirement, mode of breathing support (hourly), surfactant administration, time of SSC after birth and any cause for interrupted SSC before 120 minutes, age at the first feed (gavage or oral), any intravenous infusion, transcutaneous carbon dioxide and total amount of enteral and parenteral nutrition given. Blood pressure is measured once during the first 120 minutes.

The safety is closely monitored by the consultant and the neonatal nurse present. It will be registered as an adverse event if body temperature drops < 36.0 C° or if the infants have signs

of any respiratory failure requiring interventions in addition to an interruption of SSC and transfer to the NICU for a higher level of monitoring and/or respiratory support.

During hospitalization:

Variables recorded are: daily weight, age on removal of feeding tube, time in skin-to-skin position every day, all nutrition (parenteral and enteral), any insulin given, any surgery, mechanical ventilation (mode and duration), CPAP/BiPAP (duration), supplemental oxygen and/or ventilator support at 28 postnatal days, 36 and 40 weeks postmenstrual age (BPD) , any sepsis (with maximal C-reactive protein and duration of antibiotic), cerebral ultrasound and MRI results (IVH or PVL), seizures, NEC, treatment for PDA and post menstrual age (PMA) for transfer from intermediate to the step down unit in the NICU.

At discharge:

Variables recorded are: PMA, weight, length, head circumference and mode and type of nutrition.

Socio demographic information

Information about education, employment (partly or full time) and marital status is collected from the mothers' medical records.

Consent and enrolment

Pregnant women admitted to the maternity ward at St. Olavs University Hospital, Drammen Hospital, and Kristiansand Hospital for anticipated preterm delivery between 28^0 – 31^6 weeks of gestation are eligible. Oral and written information about the study is provided by a pediatrician and/or a neonatal nurse, and written consent is obtained before delivery,

Randomization and allocation concealment

The randomization is done after the initial stabilization of the infant. Infants are stratified by weeks of gestation (28^0-29^6 and 30^0- 31^6). The randomization is conducted using sealed envelopes organized by the Unit for Applied Clinical Research at the NTNU.

Sample size

Forty preterm infants will be included in the study. This is based on previous studies that have been able to show significant differences in evaluating care interventions in 40 preterm infants (twenty in each group) (35-37).

Ethics and approval

This study is approved by the Regional Committee of Ethics in Medical Research (Mid-Norway) (2013/638/REK midt).

References

1. Blencowe H, Cousens S, Oestergaard MZ, Chou D, Moller AB, Narwal R, et al. National, regional, and worldwide estimates of preterm birth rates in the year 2010 with time trends since 1990 for selected countries: a systematic analysis and implications. Lancet. 2012;379(9832):2162-72.
2. Lawn JE, Wilczynska-Ketende K, Cousens SN. Estimating the causes of 4 million neonatal deaths in the year 2000. International journal of epidemiology. 2006;35(3):706-18.
3. Goldenberg RL, Culhane JF, Iams JD, Romero R. Epidemiology and causes of preterm birth. Lancet. 2008;371(9606):75-84.
4. Mwaniki MK, Atieno M, Lawn JE, Newton CR. Long-term neurodevelopmental outcomes after intrauterine and neonatal insults: a systematic review. Lancet. 2012;379(9814):445-52.
5. Spittle A, Orton J, Anderson P, Boyd R, Doyle LW. Early developmental intervention programmes post-hospital discharge to prevent motor and cognitive impairments in preterm infants. Cochrane Database Syst Rev. 2012;12:Cd005495.
6. Lund LK, Vik T, Lydersen S, Lohaugen GC, Skranes J, Brubakk AM, et al. Mental health, quality of life and social relations in young adults born with low birth weight. Health and quality of life outcomes. 2012;10:146.
7. Dahl LB, Kaaresen PI, Tunby J, Handegard BH, Kvernmo S, Ronning JA. Emotional, behavioral, social, and academic outcomes in adolescents born with very low birth weight. Pediatrics. 2006;118(2):e449-59.
8. Hack M. Young adult outcomes of very-low-birth-weight children. Seminars in fetal & neonatal medicine. 2006;11(2):127-37.
9. Hack M, Youngstrom EA, Cartar L, Schluchter M, Taylor HG, Flannery D, et al. Behavioral outcomes and evidence of psychopathology among very low birth weight infants at age 20 years. Pediatrics. 2004;114(4):932-40.
10. Husby IM, Skranes J, Olsen A, Brubakk AM, Evensen KA. Motor skills at 23 years of age in young adults born preterm with very low birth weight. Early Hum Dev. 2013;89(9):747-54.
11. Saigal S, Doyle LW. An overview of mortality and sequelae of preterm birth from infancy to adulthood. Lancet. 2008;371(9608):261-9.
12. Evensen KA, Vik T, Helbostad J, Indredavik MS, Kulseng S, Brubakk AM. Motor skills in adolescents with low birth weight. Arch Dis Child Fetal Neonatal Ed. 2004;89(5):F451-5.
13. Hack M, Flannery DJ, Schluchter M, Cartar L, Borawski E, Klein N. Outcomes in young adulthood for very-low-birth-weight infants. N Engl J Med. 2002;346(3):149-57.
14. Berlin LJ, Brooks-Gunn J, McCarton C, McCormick MC. The effectiveness of early intervention: examining risk factors and pathways to enhanced development. Preventive medicine. 1998;27(2):238-45.
15. Vanderveen JA, Bassler D, Robertson CM, Kirpalani H. Early interventions involving parents to improve neurodevelopmental outcomes of premature infants: a meta-analysis. Journal of perinatology : official journal of the California Perinatal Association. 2009;29(5):343-51.
16. Mehler K, Wendrich D, Kissgen R, Roth B, Oberthuer A, Pillekamp F, et al. Mothers seeing their VLBW infants within 3 h after birth are more likely to establish a secure attachment behavior: evidence of a sensitive period with preterm infants? Journal of perinatology : official journal of the California Perinatal Association. 2011;31(6):404-10.

17. Moore KA, Coker K, DuBuisson AB, Swett B, Edwards WH. Implementing potentially better practices for improving family-centered care in neonatal intensive care units: successes and challenges. Pediatrics. 2003;111(4 Pt 2):e450-60.
18. Nuss T, Kelly KM, Campbell KR, Pierce C, Entzminger JK, Blair BK, et al. The impact of opening visitation access on patient and family experience. The Journal of nursing administration. 2014;44(7/8):403-10.
19. Nyqvist KH, Anderson GC, Bergman N, Cattaneo A, Charpak N, Davanzo R, et al. Towards universal Kangaroo Mother Care: recommendations and report from the First European conference and Seventh International Workshop on Kangaroo Mother Care. Acta Paediatr. 2010;99(6):820-6.
20. Harrison H. The principles for family-centered neonatal care. Pediatrics. 1993;92(5):643-50.
21. Als H. Toward a synactive theory of development: Promise for the assessment of infant individuality. Infant Mental Health Journal. 1982;3:229-43.
22. Als H. A Synactive Model of Neonatal Behavioral Organization: Framevork for the Assessment of Neurobehavioral Development in the Premature Infant and for Support of Infants and Parents in the Neonatal Intensive Care Environment. Physical & Occupational Therapy in Pediatrics. 1986;6 No. 3-4:3-53.
23. Nordhov SM, Ronning JA, Dahl LB, Ulvund SE, Tunby J, Kaaresen PI. Early intervention improves cognitive outcomes for preterm infants: randomized controlled trial. Pediatrics. 2010;126(5):e1088-94.
24. Nordhov SM, Ronning JA, Ulvund SE, Dahl LB, Kaaresen PI. Early intervention improves behavioral outcomes for preterm infants: randomized controlled trial. Pediatrics. 2012;129(1):e9-e16.
25. WHO/UNICEF. Baby-friendly hospital initiative : revised, updated and expanded for integrated care. 2009 [Available from: http://www.who.int/nutrition/publications/infantfeeding/bfhi_trainingcourse/en/. Accessed 18 Sept 2015.
26. Bergman NJ, Linley LL, Fawcus SR. Randomized controlled trial of skin-to-skin contact from birth versus conventional incubator for physiological stabilization in 1200- to 2199-gram newborns. Acta Paediatr. 2004;93(6):779-85.
27. Chi Luong K, Long Nguyen T, Huynh Thi DH, Carrara HP, Bergman NJ. Newly born low birthweight infants stabilise better in skin-to-skin contact than when separated from their mothers: a randomised controlled trial. Acta Paediatr. 2015.
28. Morelius E, Ortenstrand A, Theodorsson E, Frostell A. A randomised trial of continuous skin-to-skin contact after preterm birth and the effects on salivary cortisol, parental stress, depression, and breastfeeding. Early Hum Dev. 2015;91(1):63-70.
29. World Health Organization. WHO Recommendations on Interventions to Improve Preterm Birth Outcomes Geneva: World Health Organization; 2015 [Available from: http://apps.who.int/iris/bitstream/10665/183037/1/9789241508988_eng.pdf?ua=1. Accessed 15 Des 2015.
30. Kristoffersen L, Stoen R, Hansen LF, Wilhelmsen J, Bergseng H. Skin-to-Skin Care After Birth for Moderately Preterm Infants. J Obstet Gynecol Neonatal Nurs. 2016.
31. Bayley N. Bayley Scales of Infant and Toddler Development. San Antonio: Harcourt Assesment; 2005; 2005.
32. Einspieler C, Prechtl HF, Bos AF, Ferrari F, Cioni G. Prechtl's method on the qualitative assessment of general movements in preterm, term and young infants. New York: Mac Keith Press; 2004.
33. Ferrari F, Cioni G, Prechtl HF. Qualitative changes of general movements in preterm infants with brain lesions. Early Hum Dev. 1990;23(3):193-231.
34. Squires J, Bricker D, Heo K, Twombly E. Ages & Stages Questionnaires: Sosial-Emotional (ASQ:SE) A parent-completed, child-monitoring system for social-emotional behaviors.: Baltimore: Brookes; 2002.
35. Catelin C, Tordjman S, Morin V, Oger E, Sizun J. Clinical, physiologic, and biologic impact of environmental and behavioral interventions in neonates during a routine nursing procedure. J Pain. 2005 Dec;6(12):791-7.
36. Sizun J, Ansquer H, Browne J, Tordjman S, Morin JF. Developmental care decreases physiologic and behavioral pain expression in preterm neonates. J Pain. 2002 Dec;3(6):446-50.
37. Kleberg A, Warren I, Norman E, Morelius E, Berg AC, Mat-Ali E, et al. Lower stress responses after Newborn Individualized Developmental Care and Assessment Program care during eye screening examinations for retinopathy of prematurity: a randomized study. Pediatrics. 2008 May;121(5):e1267-78.

2. Summary of main amendments to the original study protocol

Domain	Original protocol	Revised to/added	Rationale
Sample size and analysis plan	No formal sample size calculations were performed. Inclusion of 40 very preterm infants (20 in the skin-to-skin group and 20 in standard care was described based on previous studies that have been able to show significant differences in evaluating care interventions.	Sample size calculations were performed for a two-sample t-test comparing the Bayley Scale of Infant and Toddler Development, third edition, cognitive score between the intervention (skin-to-skin care, SSC) and control (standard care, SC) groups at two years corrected age. To obtain a power of 80% for detecting a difference of 7.5 in mean score, using SD=15 and significance level α=0.05, 64 preterm infants are needed in each group. To allow for withdrawals, the sample size was set to 68 in each group.<br><br>*Date of change: February 2016*	A proper sample size calculation was conducted because the initial description of infants to be included was not enough to reveal any differences between the groups.
Cooperation	Only one hospital in Norway were recruiting participants (St. Olav's Hospital)	Cooperation with two additional hospitals in Norway from January 2017 (Drammen Hospital and Sørlandet Hospital, Kristiansand)<br><br>*Date of change: October 2016*	Fewer participant than expected were included during the first 2 years of enrollment. Cooperation will increase the number of participants and enable the sample size to be reached
Outcomes within 24 hours after birth	1. Measurement of the premature child's stress response from saliva within the first 24 hours after birth<br><br>2. Video recording of infants preterm general movements (Preterm GMA)	Removed from protocol.<br><br>Removed from protocol.<br><br>*Date of change: February 2016*	Difficult to collect enough saliva for a valid measurement<br><br>Difficult to coordinate and is therefore not considered feasible.
Outcomes at 1 year	None planned	Two outcomes were added, of which one was changed during the study<br>1. Neurodevelopmental outcomes at 1 year<br>a. Bayley Scale of Infant and Toddlers Development, third edition (BSID-III)	1a. We wanted to add an evaluation of neurodevelopmental outcomes between 3 months and 2 years of age

		Date of change: May 2015<br><br>b. Ages & Stages Questionnaire (ASQ) and ASQ: Social and Emotional (ASQ-SE)<br><br>*Date of change: April 2017*	 <br>1b. After 2 years of enrollment, the 12-month BSID-III was replaced with the ASQ and ASQ-SE due to limited staff resources to conduct the testing and to reduce the burden on the families.
		2. Nutrition (breastfeeding/breastmilk) up to 12 months of age.<br><br>*Date of change: June 2017*	2. The intervention had been described as favorable for breastfeeding in former publications. We therefore wanted to collect data on nutrition in our study. Data were collected retrospectively for those already included and prospective for the rest.

3. Summary of amendments to the published study protocol

Domain	Original protocol	Revised to/added	Rationale
Cooperation		A cooperation with IWK Health Centre, Halifax, Canada.<br><br>*Date of change: June 2019*	Because fewer participants than expected were included in Norway until 2019, a collaboration with Canada was prepared, formalized, and initiated. However, difficulties with recruitment and implementation in Canada made it impossible. No infants were therefore included.

Region:	Executive officer:	Phone:	Our date:	Our reference:
REK midt	Hilde Eikemo	+004773597508	February 6, 2020	2013/638/REK midt

To whom it may concern,

The Regional Committee for Medical and Health Related Research Ethics, REC Central Norway, hereby confirm that the project "Skin-to-skin after delivery in preterm infants born at 28+0 - 31+6 weeks of gestation. A randomized controlled trial. " with Laila Kristoffersen as the principal investigator, has been evaluated and approved by the committee. The study was originally evaluated by the committee in its meeting on 19 April 2013. The committee had a few remarks to the protocol, and asked for a revision. The project was subsequently approved on 11 October 2013.
Since then, several amendments have been made to the protocol and they have all been approved by REC. The last amendment was approved on 25 June 2019.

Sincerely,

Vibeke Videm
Professor, MD, PhD
Chairman of the Committee

Hilde Eikemo
Head of Secretariat
