# Supplementary material for: Immediate Skin-to-Skin Contact in Very Preterm Neonates and Early Childhood Neurodevelopment: A Randomized Clinical Trial
Source: JAMA Netw Open. 2025 Apr 16;8(4):e255467. doi: 10.1001/jamanetworkopen.2025.5467 (PMC12004208; doi:10.1001/jamanetworkopen.2025.5467)
Supplement: Supplement 2. — eFigure. Overview of Outcome Assessments at 3 and 12 Months and 2 to 3 Years of Corrected Age eTable 1. Maternal and Neonatal Characteristics Among Infants With the Primary Outcome, According to Intervention Group eTable 2. Neurodevelopmental Outcomes at 3 and 12 Months of Corrected Age eReferences. [file jamanetwopen-e255467-s002.pdf]

Supplementary Online Content

Kristoffersen L, Støen R, Bergseng H, et al. Immediate skin-to-skin contact in very preterm neonates and early childhood neurodevelopment: a randomized clinical trial. *JAMA Netw Open*. 2025;8(4):e255467. doi:10.1001/jamanetworkopen.2025.5467

eFigure. Overview of Outcome Assessments at 3 and 12 Months and 2 to 3 Years of Corrected Age
eTable 1. Maternal and Neonatal Characteristics Among Infants With the Primary Outcome, According to Intervention Group
eTable 2. Neurodevelopmental Outcomes at 3 and 12 Months of Corrected Age
eReferences.

This supplementary material has been provided by the authors to give readers additional information about their work.

© 2025 Kristoffersen L et al. *JAMA Network Open*.

eFigure. Overview of Outcome Assessments at 3 and 12 Months and 2-3 Years of Corrected Age

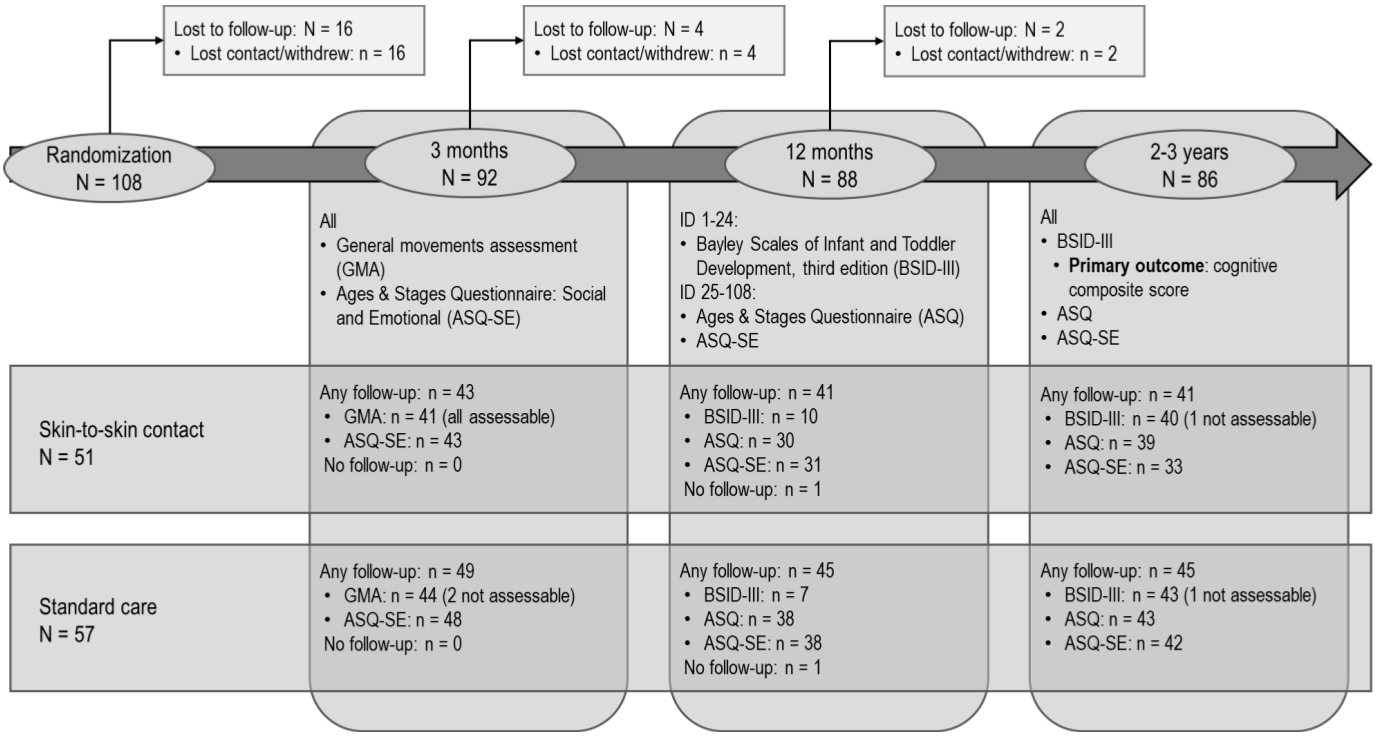

© 2025 Kristoffersen L et al. *JAMA Network Open*.

eTable 1. Maternal and Neonatal Characteristics Among the Infants with Primary Outcome According to Intervention Group

	**Skin-to-skin contact**		**Standard care**	
**Maternal characteristics**	**Total No.**		**Total No.**	
Maternal age at delivery, mean (SD), y	36	32.5 (4.4)	40	29.9 (5.9)
University education, No. (%)[a]	36	27 (75)	39	22 (56)
Cesarean delivery, No. (%)	36	21 (58)	40	24 (60)
Multiple birth/twins, No. (%)	36	3 (8)	40	2 (5)
**Neonatal characteristics**	**Total No.**		**Total No.**	
Gestational age, mean (SD), wk	39	30.3 (1.1)	42	30.3 (1.1)
Birth weight, mean (SD), g	39	1435 (246)	42	1441 (268)
Sex, No. (%)				
Male	39	23 (59)	42	27 (64)
Female	39	16 (41)	42	15 (36)
Growth, No. (%)[b]				
Small for gestational age	39	0	42	1 (2)
Large for gestational age	39	2 (5)	42	2 (5)

[a] Completed education at bachelor’s level or higher.
[b] Growth was calculated by PediTools Growth Parameters based on the 2013 Fenton growth charts. Small for gestational age is defined as birth weight below the 10th percentile and large for gestational age as birth weight above the 90th percentile[1,2]

© 2025 Kristoffersen L et al. *JAMA Network Open*.

eTable 2. Neurodevelopmental Outcomes at 3 and 12 Months of Corrected Age

	**Skin-to-skin contact**		**Standard care**		**P value**
**3 months**	**Total No.**		**Total No.**		
General movement assessment [a]					
Absent fidgety movements, No. (%)	41	0 (0)	42	1 (2)	1.000
Movement Optimality Score-Revised (MOS-R), median (IQR)	41		42		
Total score		26 (24-28)		26 (24-28)	0.981
Total score without fidgety movement		14 (12-16)		14 (14-16)	0.934
ASQ-SE[b]					
Total score, median (IQR)	43	15 (5-30)	43	20 (10-35)	0.185
**12 months**					
BSID-III					
Cognitive composite score, mean (SD)	10	94.5 (11.9)	7	94.3 (8.9)	0.968
Language composite score, mean (SD)	10	90.1 (12.9)	7	95.6 (13.9)	0.417
Motor composite score, mean (SD)	10	94.6 (6.8)	7	91.4 (14.5)	0.552
ASQ[c]					
Gross motor, median (IQR)	30	45 (30-50)	38	50 (35-60)	0.272
Fine motor, median (IQR)	31	50 (45-60)	38	50 (45-60)	0.656
Problem-solving, median (IQR)	30	48 (35-55)	38	45 (35-55)	0.925
Communication, median (IQR)	30	38 (25-50)	38	40 (25-50)	0.704
Personal/social, median (IQR)	30	35 (30-45)	38	45 (40-55)	0.021
ASQ-SE[b]					
Total score, median (IQR)	31	20 (13-40)	38	15 (10-32)	0.391

Abbreviations: ASQ-SE, Ages and Stages Questionnaire-Social Emotional; BSID-III, Bayley Scales of Infant and Toddlers Development third edition; ASQ, Ages and Stages Questionnaire.

[a] 2 infants had general movement assessments that were not assessable for fidgety movement and MOS-R score.

[b] The lower the score the better

[c] The higher the score the better. One had only data on fine motor skills

© 2025 Kristoffersen L et al. *JAMA Network Open*.

eReferences

1. Chou JH, Roumiantsev S, Singh R. PediTools Electronic Growth Chart Calculators: Applications in Clinical Care, Research, and Quality Improvement. *J Med Internet Res*. Jan 30 2020;22(1):e16204. doi:10.2196/16204

2. Fenton TR, Kim JH. A systematic review and meta-analysis to revise the Fenton growth chart for preterm infants. *BMC Pediatr*. Apr 20 2013;13:59. doi:10.1186/1471-2431-13-59

© 2025 Kristoffersen L et al. *JAMA Network Open*.
