# Supplementary material for: Immediate Skin-to-Skin Contact in Very Preterm Neonates and Early Childhood Neurodevelopment: A Randomized Clinical Trial
Source: JAMA Netw Open. 2025 Apr 16;8(4):e255467. doi: 10.1001/jamanetworkopen.2025.5467 (PMC12004208; doi:10.1001/jamanetworkopen.2025.5467)
Supplement: Supplement 3. — Data Sharing Statement [file jamanetwopen-e255467-s003.pdf]

Data Sharing Statement

Kristoffersen. Immediate Skin-to-Skin Contact in Very Preterm Neonates and Early Childhood Neurodevelopment. *JAMA Netw Open*. Published April 16, 2025.
doi:10.1001/jamanetworkopen.2025.5467

Data
Additional Information: https://clinicaltrials.gov/study/NCT02024854
Data available: No

Additional Information
Explanation for why data not available: Data sharing is not applicable for this study as data was collected before January 2019 and are not compatible with the Norwegian Health research act and privacy.
